# Key Decision Points for Personalization and Customization in Self-Guided Digital Suicide Prevention: Qualitative Interview Study

**DOI:** 10.2196/95930

**Published:** 2026-10-14

**Authors:** Zhiying Quan, Catherine Chen, Sarah A Popowski, Theresa Nguyen, Kevin Rushton, Madhu Reddy, David C Mohr, Kimberly A Van Orden, Jonah Meyerhoff

**Affiliations:** 1 Feinberg School of Medicine, Northwestern University, 750 N. Lake Shore Dr. 10th Floor, Chicago, IL, 60611, United States, 1 312-503-1232; 2 Mental Health America, Alexandria, VA, United States; 3 Department of Informatics, Donald Bren School of Informatics and Computer Science, University of California, Irvine, CA, United States; 4 Department of Psychiatry, University of Rochester Medical Center, Rochester, NY, United States

**Keywords:** personalization, customization, decision points, digital suicide prevention, digital safety planning intervention, digital mental health interventions

## Abstract

**Background:**

Young adults in the United States experience high rates of suicidal thoughts and behaviors (STB) but low engagement with traditional care, highlighting the need for accessible and scalable digital interventions. Self-guided digital safety planning interventions (SPIs) can expand access to support but introduce important decisions about what content to deliver, when systems should intervene, and how users retain control over system actions. Personalization, defined as system-initiated adaptation, and customization, defined as user-initiated control, may offer complementary approaches to these decisions. However, how they should be coordinated to preserve autonomy and reduce burden during periods of distress remains unclear.

**Objective:**

This study aims to identify key decision points in self-guided digital SPIs and characterize what, when, how, and by whom (system or user) safety planning functions should be controlled. We further examined how personalization and customization can jointly support autonomy, reduce burden, and promote safety.

**Methods:**

Nine young adults (ages 18‐24) with lived experience of suicidal thoughts and behaviors were recruited from an asynchronous remote community comprising individuals who had engaged with the Mental Health America (MHA) website. Participants completed semistructured individual interviews via Zoom. Three coders analyzed interview transcripts using codebook thematic analysis. One coder open-coded all transcripts to develop a preliminary codebook. Two additional coders then applied the codebook to overlapping transcripts, after which all coders revised the codebook and resolved discrepancies by consensus before coding the remaining transcripts. Themes were interpreted using the 5W1H (Who, What, Where, When, Why, and How) framework as an organizing lens for identifying and structuring decision points.

**Results:**

Participants identified 3 classes of decision points for self-guided digital SPIs: (1) system-level decisions about what content and actions are automatically provided; (2) user-level decisions about when and at what pace safety planning unfolds; and (3) shared decisions requiring coordination between users and the system. Participants favored system personalization of decision aids, coping suggestions, brainstorming prompts, example messages for contacting trusted others, affirmations, and relevant resources. In contrast, participants wanted control over the timing and pacing of safety planning and the representation and organization of their safety plans. Participants also emphasized that systems should obtain explicit permission before taking safety-relevant actions, such as contacting trusted others or connecting users with crisis counselors in response to increases in detected risk.

**Conclusions:**

Personalization and customization can function as complementary mechanisms for allocating decision authority within self-guided digital SPIs. The findings support a model of permissioned automation in which systems reduce cognitive and emotional burden through adaptive support while users retain control over safety-relevant actions. This decision-point framework provides a foundation for designing and evaluating self-guided suicide prevention technologies that balance adaptation, autonomy, and safety.

## Introduction

A widening discrepancy between the elevated suicide risk among young adults and the low use of mental health services indicates a critical need for research and interventions. Suicide is one of the leading causes of death in the United States. In 2024, 14.3 million (5.5%) of adults (aged 18 or older) had serious thoughts of suicide within the past year, and 2.2 million attempted suicide [1]. Suicide prevention interventions are often delivered through formal mental health care settings; however, over 41% of adults who endorse a need for mental health treatment do not receive treatment [1]. These data suggest that traditional service delivery models are not sufficiently reaching this population.

The safety planning intervention (SPI) is considered a best practice [2,3] in behavioral health settings when working with individuals who have elevated suicide-related thoughts or behaviors. The SPI was developed as a brief and collaborative clinician-delivered intervention [4] that aims to support patients in coping with suicidal thoughts and resisting suicidal behaviors by creating a highly personalized list of internal and external coping resources [5]. The SPI often comprises 7 steps: (1) identifying warning signs, (2) coping strategies, (3) people who can provide distraction, (4) trusted contacts to help in a crisis, (5) professional resources, (6) lethal means safety, and (7) reasons for living. It also includes follow-up contact from providers to support connections to care and support using one’s safety plan [4]. However, young adults face structural and attitudinal barriers, such as cost, stigma, and limited access to providers, that prevent them from engaging in formal mental health treatment [6,7]. Moreover, this clinician-delivered intervention may face demand barriers, such as young adults’ strong preferences for self-reliance and autonomy [8,9].

Digital SPIs offer a potential pathway to circumvent these barriers by offering low-burden, accessible, and scalable interventions via mobile platforms [10,11]. As digital versions of SPIs have expanded, it is important to distinguish between collaborative SPIs completed jointly with clinicians through telehealth platforms and self-guided SPIs completed independently with structured system directions and guidance [12]. Emerging randomized controlled trials comparing in-person and digital SPIs suggest that some self-guided and interactive digital SPIs are acceptable, feasible, and yield effective safety plans that rival clinician-delivered safety plans [12,13]. Digital SPIs have been developed across various platforms and populations, with many offering self-guided formats designed to help individuals manage suicidal crises [14-18]. These self-guided safety plans demonstrate acceptability and show promise for increasing the accessibility of SPIs.

Within self-guided digital SPIs, there are decision points that must be considered as part of their design, specifically how users are expected to engage with safety planning tools and the level of decisional authority they can retain throughout the planning process. We organize these decision points using the 5W1H (Who, What, Where, When, Why, and How) framework, a structure used in human-centered design work to identify and organize decision points in complex behavioral interventions [19]. In this study, *what*, *when*, and *how* organize the primary decision-point domains, while *who*, *where*, and *why* function as contextual conditions that cut across those domains, grounding the needs-assessment logic that shapes how each decision point should be operationalized.

The current study adopts a clear conceptual distinction between personalization and customization as 2 mechanisms through which control over functionality and decision support can be implemented in a self-guided digital SPI. Personalization typically involves the system automatically tailoring content to users based on user data, while customization involves users actively choosing and modifying the interface or content [20,21]. In this study, personalization refers to system-initiated adaptation, in which the intervention automatically tailors features of, or content within, a system, while customization refers to user-initiated control that defines when, what, and how users can actively make choices about their experience using a system, increasing user autonomy and engagement [20,22]. Prior research identifies both personalization [23] and customization [24] as facilitators of engagement with digital mental health interventions (DMHIs) among young people.

To understand how both personalization and customization can be applied in self-guided digital SPIs, we conducted one-on-one interviews with young adults with lived experience of suicidal thoughts and behaviors (STB). We aimed to answer the following research questions: (1) what are the key decision points in digital SPIs? (2) How do personalization and customization function as mechanisms for allocating autonomy and decision authority across safety planning decision points?

## Methods

### Participants

Nine young adults who participated in a larger asynchronous remote community (ARC) study about the design of text-based, self-guided digital safety plans [25] provided affirmative informed consent to participate in individual interviews to understand their needs and preferences regarding digital safety planning (Table 1). Participants were recruited by email if they had participated in the parent study [25]. Eligibility criteria for the parent study required participants to reside in the United States, be between 18 and 24 years old, own a smartphone, possess proficiency in English, and report suicidal ideation within the 2 weeks prior to recruitment. Suicidal ideation was assessed using a modified item from the *DSM-5*-CCSI (*Diagnostic and Statistical Manual of Mental Disorders* [Fifth Edition] Cross-Cutting Symptom Inventory [26]), the DSI-SS (Depression Severity Index–Suicidality Subscale [27]), and an item (#9) from the BDI-II (Beck Depression Inventory–Second Edition [28]). Exclusion criteria included manic or psychotic symptoms endorsed within the 2 weeks prior to recruitment or the presence of strong suicidal desire, operationalized as a score of 2 or greater on BDI-II item 9.

**Table 1.** Participant characteristics (N=9).

Characteristics	Total
Age (y), mean (SD)	21.7 (2.1)
Gender, n (%)	
Female	7 (78)
Nonbinary	2 (22)
Transgender, n (%)	2 (22)
Race, n (%)	
Asian	2 (22)
White	6 (67)
More than 1 race	1 (11)
Hispanic/Latino, n (%)	2 (22)
DSI-SS^a^ total, mean (SD)^b^	5.2 (0.7)
Ever seen a therapist or mental health professional, n (%)	8 (89)
Currently seeing a therapist or mental health professional, n (%)	1 (11)
Plan to see a therapist or mental health professional in the next 8 weeks, n (%)	6 (67)

a DSI-SS: Depression Severity Index–Suicidality Subscale.

b Four items each coded 0 to 3; range 0 to 12, where higher scores mean more intense suicidal thoughts.

### Procedures

The parent project examined (1) the overall needs and preferences of young adults with STB for suicide prevention and (2) the needs and preferences young adults had for a SPI delivered via interactive text messaging [25]. Participants were recruited for the parent project through an advertisement embedded on Mental Health America’s (MHA) screening website, where they host free, validated screening tools [29]. Individuals early in their mental health journeys go to learn more about their mental health symptoms and plan next steps [30]. The advertisement was shown to individuals who accessed a resource page about STB and reported recent suicidal or self-harm ideation on one of MHA’s online mental health screening tools. Interested individuals who clicked on the advertisement were directed to a Northwestern University–hosted study information page. Those wishing to participate could complete eligibility screening surveys via Northwestern’s REDCap (Vanderbilt University) [31,32]. Eligible individuals reviewed informed consent documentation and provided affirmative informed consent prior to any study procedures.

Participants (N=9) in this interview study were recruited from a pool of individuals who participated in one of 2 ARC groups [25] that explored the needs and preferences of young adults who have experienced STB as part of the parent research project. The parent ARC study included 30 participants, with 15 participants in each of the 2 groups. Interview invitations were sent to 26 of the 30 ARC participants, prioritizing those who had made at least one ARC post. The 26 invitees included 2 participants who had not engaged in ARC activities, whom we invited to capture potential perspectives from individuals who may have disengaged from the ARC forum; neither responded. Four ARC participants were not invited because they had no engagement with ARC activities; one of these participants had previously withdrawn from the study. Of the 26 participants invited, 9 scheduled and completed an interview, 1 declined, and 2 reported scheduling conflicts that could not be resolved and did not respond to additional scheduling options. The remaining 14 participants did not respond to multiple outreach attempts.

These 9 individual interviews were conducted by the principal investigator, a doctoral-level clinical psychologist, or a full-time research assistant with a bachelor’s degree. The interview guide was drafted by the study principal investigator, JM, drawing on interview guides used in the team’s prior research, the aims of the current study, and his experience in suicide prevention, digital mental health, and qualitative research. MHA collaborators (TN and KR) also reviewed the guide to ensure that it was appropriate for young adults seeking mental health information through the MHA platform. The guide also aimed to elicit additional details and clarifications beyond what participants shared in their ARC elicitation groups. Data for the present study comprise transcripts from 9 individual interviews. Interviews were conducted via Zoom, with camera use optional. Interviews were conducted by JM and SAP. SAP had communicated with participants about study logistics. Neither of the 2 interviewers had a preexisting personal or clinical relationship with participants. Both interviewers were familiar with techniques for eliciting detailed responses while remaining neutral and maintaining fidelity to the semistructured interview guide. Interviews lasted approximately 20 to 40 minutes, and participants received US $8 for completing the interview. This sample size of 9 reflects the number of ARC participants who completed the follow-up interview.

### Reflexivity Statement

The research team included 9 authors with diverse backgrounds and levels of experience, including practicing clinicians, behavioral health researchers, mental health professionals, and social science students. The team represented a range of educational and professional backgrounds, including psychology, social work, digital mental health, communications, human-computer interaction, and computer science, as well as career stages ranging from early-, middle-, and advanced-career researchers and staff to doctoral-level faculty members and trainees, including master’s degree students. Collectively, the team has a strong interest in the development and evaluation of digital mental health tools and interventions and in increasing access to suicide prevention care. The team is aware of its predisposition toward the potential of accessible digital suicide prevention tools to increase access to mental health care.

At the time of the study, JM was a clinical psychologist and faculty member with clinical and research experience in suicide prevention. JM was leading funded work focused on increasing access to care through the design, development, and evaluation of DMHIs. SAP was a research assistant who helped administer the ARC activities and communicated with participants about study logistics. ZQ was a master’s student working with JM and had no prior contact with participants. The team remained attentive to how these differences in roles, experiences, and levels of participant contact could shape the data collection and interpretation. The team actively engaged in discussions and reflected on their assumptions and perspectives to ensure that interpretations were grounded in participants’ accounts.

### Data Analysis

All interviews were audio-recorded and transcribed by a contracted human transcription service and were reviewed for accuracy by the transcriber. Each transcript was analyzed using codebook thematic analysis [33] with the qualitative analysis management software Dedoose (Sociocultural Research Consultants, LLC [34]). The lead author (ZQ) open-coded all 9 interview transcripts to develop a preliminary codebook. Two additional coders (JM and SAP) each applied the preliminary codebook to 2 overlapping transcripts. All 3 coders then met to discuss their interpretations, refine the code definitions, review, and further refine the codebook. The team resolved discrepancies through discussion and consensus. Following the meeting, the team developed a revised codebook agreed upon by all coders. The revised codebook was then applied across the remaining transcripts, and the lead author (ZQ) reviewed the fully coded dataset for consistency. No formal interrater reliability statistic was calculated. After coding was completed, related codes and coded excerpts were organized into broader patterns concerning system-level decisions, user-level decisions, and interactions between system guidance and user control. The 5W1H framework was subsequently applied as an interpretive and organizational lens to clarify and communicate the identified decision points.

Several features of the analytic process supported the trustworthiness of the findings. Credibility was supported through review of overlapping transcripts by multiple coders, discussion of differing interpretations, refinement of the codebook, and use of participant quotations to support the findings. Dependability was supported through the use of a documented codebook process and the retention of records of codebook development and analytic discussions. Confirmability was supported through review of the full coded dataset by the lead coder, team discussion of interpretations, and grounding of the reported findings in coded excerpts and participant quotations. To help readers assess transferability, we provide detailed information about the MHA recruitment context, the parent ARC study, eligibility criteria, and participant characteristics.

Due to the nature of the semistructured follow-up interviews, where some interview questions were tailored based on participants’ responses in the initial ARC workshops, coding was conducted at the level of a paired interviewer question and participant response. This ensured that the context of the interviewer’s question was included in the analysis. Each time a part of the interview matched the criteria for a specific theme, we applied the corresponding code to that segment.

### Ethical Considerations

All study procedures were reviewed and approved by the Northwestern University Institutional Review Board (STU00217191). Before completing any study procedures, participants provided affirmative informed consent for participation in the parent study and the follow-up interview. Participants also consented to multimedia recording of the interview and the use of deidentified interview data and quotations in research publications. Participants received US $8 for completing the interview.

Interviews were conducted via Zoom and audio-recorded. Recordings were transcribed by a contracted human transcription service and reviewed for accuracy by the transcriber. Interview recordings and transcripts were kept as confidential. Transcripts were deidentified and stored on secure, encrypted servers maintained by Northwestern University’s Feinberg School of Medicine. Transcripts were redacted by research staff to remove any potentially identifying information (eg, names). Audio recordings were archived on encrypted Feinberg School of Medicine servers in accordance with the approved protocol and will be deleted following study closure.

Because participants had lived experience of STB, interviewers followed an institutional review board–approved safety protocol when disclosures indicated potentially elevated suicide risk. The protocol included a brief risk assessment using the Columbia Suicide Severity Rating Scale (C-SSRS) [35] and risk-responsive follow-up procedures, which could include developing a coping plan or facilitating a connection to the 988 Suicide & Crisis Lifeline. All interview personnel were trained in these procedures before conducting interviews. The safety protocol was not activated during any of the interviews.

## Results

### Decision Points for Personalization and Customization in Digital SPIs

Participants in our study identified 3 classes of decision points for self-guided digital SPIs: (1) system-driven personalization, where the intervention automatically adapts by delivering tailored suggestions, resources, or messages based on user data or contextual cues; (2) user-driven customization, where individuals actively adjust content, pacing, or features to suit their preferences and needs; (3) the interaction between personalization and customization, where users navigate between automated guidance and personal control. These decision points provide valuable insights into how self-guided digital SPIs can be designed to better meet the needs of young adults during crisis moments.

In the sections that follow, participant quotation labels indicate the ARC wave (W1 or W2) and a coded participant number (P).

### What Elements of Self-Guided Digital Safety Planning Do Young Adults Want Personalized?

Below, we detail ways in which participants discussed the value of a digital SPI that personalizes aspects of safety planning using decision trees or algorithms.

#### System Suggestions, Guidance, and Support

Participants emphasized the value of receiving suggestions, guidance, and emotional support from the system, especially when they experienced overwhelming emotions, uncertainty, or difficulty practicing parts of their safety plan.

Across interviews, participants shared that they were interested in having automated decision aids that informed them about professional, supportive people or organizations they could ask for help if a crisis was escalating. For instance, W1-P2 noted how professional connections offered by the system can be helpful, “If the automated system recognizes signs of imminent suicidality in me, if it said something like, ‘I’m noticing that you’re deep in crisis’ or ‘I’m noticing that you’re having some pretty intense warning signs. Would you like to be connected to a trained crisis counselor?’” Participants further expressed the need for the system to inform a decision regarding when they should actually contact supportive individuals. W2-P9 described that, “I can imagine it maybe prompting you to talk to someone. For example, if you’re working on this tool, and you’re expressing that you’re kinda struggling right now, and you need a resource, or you to need some kind of help. I wonder if it could prompt you to have you reach out to this friend yet.”

Participants also valued prompts that fostered idea generation or brainstorming of coping strategies. Participants noted that this would be especially useful when building lists of warning signs, safe, trusted others to contact, and coping skills. The process of brainstorming needed to feel individualized based on the participants’ own experiences. As W2-P9 imagined that, “I guess that’s what I’m trying to say is if it were to suggest things based on your own experience it could kind of guide you through that process of identifying what you’re experiencing.” To accomplish this, 1 participant emphasized the importance of open-ended, reflective prompts that could elicit meaningful input and encourage deeper engagement:

*Instead of, like, “Does this make you feel sad,” or something…definitely open-ended, helping me think about all the possibilities. “What are some warning signs you have noticed that might lead to crisis? How do they make you feel? How do you think they affect other people? How have you noticed them affecting your mood? Your day? Your appetite? How do they affect all of this in your life?” Instead of just asking “what” questions, asking “how” questions and “why/when” questions. Things that make me need to write more than just one or two words.*

[W1-P2]

Another element of self-guided digital safety planning that participants thought would be useful to personalize was generating example messages one could send to trusted others if they needed a distraction or help staying safe. Contacting trusted others for distraction and help is a key step in the safety planning process. However, knowing what to say to these individuals to elicit the required distraction or help can be difficult in a crisis state. Therefore, participants noted that having example messages or scripts they could adapt or use would be helpful for initiating difficult conversations about STB (eg, disclosing distress to a trusted other). When the interviewer asked whether the participant was interested in modifiable example text messages for reaching out, “How much would you be interested in language suggestions that you could then modify? Texts that you could then modify like, ‘Here is how to start a conversation with somebody that you were wanting to. Here’s a text message with some example language for how to open a conversation around this.’” Participant W2-P5 responded, “Yeah, I’d be interested in something like that. Sure.” Some participants reflected on the utility of a system suggesting script language that could facilitate help-seeking. Participant W2-P9 stated, “It could give you that prompt to reach out to someone, and then it could optionally offer a prompt. Cause that could be definitely helpful for sure.”

Participants emphasized the importance of personalizing the delivery of automated affirmations following the completion of safety planning sections, particularly when they felt unsure about how to complete certain parts of the safety plan. One participant, W1-P3, shared that when generating aspects of the safety plan, they are often uncertain. Having automated affirming messages could build self-confidence and suicide-related coping confidence: “there are some things in there that I can’t even answer right now cause I don’t even have the answers to. So, like I was just thinking maybe something that could be there that’s kind of like, ‘Oh, it’s okay. Don’t panic yet,’ kind of like a little reassurance…maybe just trying to relieve some pressure.”

Other participants explained that system-initiated positive affirmations could help alleviate the burden of reaching out for help, further reinforcing help-seeking as a continuous, positive habit:

*…for me, I don’t reach out to people until I hit rock bottom because I’m convinced that I can just fix it before I get there. And if I reach out to someone asking for help, it’s like I’ve failed again. And if the system could have some way of providing positive affirmations of, “It’s not gonna be linear. You’re doing great. Look how great you’ve been doing because you’ve been texting us this often and this many times in the last month or so.” “Or you texting us is the first step, and congratulations.” Right? Because the goal is to encourage someone to keep coming back, and to keep reaching support at earlier, and earlier stages.*

[W2-P6]

#### Personalized Support Resources

Participants emphasized the importance of receiving personalized, trustworthy, and relevant coping resources from the system, spanning crisis helplines, online tools, and other external coping resources.

Participants shared that they wanted a digital SPI to automatically identify and provide them with lists of localized resources based on the user-provided location information. Localized resources were viewed as useful because they are more accessible and may offer services that are more relevant to individuals’ needs based on their location. As 1 participant explained:

*Being able to tell them where you live. And if they could compile a list like that or even direct them to their local mental health authority so that they can get resources from them …And being able to direct them to a link or something with a list of those resources.*

[W1-P2]

This participant highlighted a desire for the system to automatically tailor mental health resources based on their geographic information. They shared how regional resources could enhance the relevance of the services for individuals who experience STB, as well as help them prepare more effectively for moments of distress by providing knowledge of the available, geographically tailored mental health service infrastructure.

In general, participants emphasized the importance of comprehensive and diverse resource provision within a self-guided digital safety planning tool that offers users information about services across different levels of need, spanning crisis resources, emergency care, and everyday well-being support. Participants valued crisis resources such as crisis helplines and text lines, noting that the intervention system could automatically organize and present the relevant resource to reduce cognitive burden during moments of distress. Other participants expressed interest in noncrisis supports such as warmlines, which extend care beyond crisis moments and promote overall well-being while fostering ongoing everyday support. In addition, some participants stressed the need for clarity about available resources, particularly emergency care. For example, W1-P1 noted confusion about emergency care options and what to expect, explaining, “it’s sad, I should know more about this [emergency departments]…with the place where you end up staying for like a week when stuff goes really shitty.”

### How Do Young Adults Want to Actively Customize and Control Self-Guided Digital Safety Planning?

Below, we highlight participants’ discussions of digital safety planning elements and the decisions over which participants preferred to maintain full control.

#### Timing, Pacing, and Responsiveness as User-Controlled Decision Points

Rather than relying on fixed schedules or uniform approaches, participants preferred tools that gave them a choice over when, how, and how much to engage, especially during moments of emotional overwhelm or executive dysfunction. Participants expressed a desire to set their own latency for responding—the time allowed for a user to respond to a message or prompt within the intervention before the system automatically advances. Participants shared that, especially as it related to a text message–delivered intervention, variation in energy levels and motivation could become a barrier to meaningful engagement in creating a digital safety plan. By controlling the response latency allowed, participants could adjust the message or prompt cadence according to their energy and motivation levels. One participant noted the ability to respond depending on a user’s motivation or energy level. As W2-P7 articulates, “with text messages you have more of a choice with how long it takes for you to respond, and whether you do respond.” Others echoed this idea and described that a substantial message latency could be especially helpful for supporting continued motivation to use the intervention. As W2-P8 put it, “...letting the system know you need more time...my first thought was one day needs to pass, but I can see the benefits of eight hours. I feel like that seems like it’s enough time for maybe something to happen in between those messages for a response.”

Relatedly, participants had varied preferences for the cadence of safety planning. Some preferred completing safety plans in 1 sitting, offering a sense of closure and continuity, while others appreciated the option to work through it section by section across multiple days. As W1-P4 explained, comorbid symptoms made long tasks difficult, so they desired the option to break safety planning into smaller, less overwhelming segments.

*I feel like the hour that you mentioned might be the longest that I’d want to have to spend on it…But I feel like you being able to complete it in a shorter period of time, maybe 15 or 20 minutes…might make it more accessible…with the messages in general, if it’s longer, it might be harder to take in all that information, or I might procrastinate because my ADHD… But having it split up into smaller pieces would definitely help even if it took longer, having it in clear segments that I can complete in a set amount of time would definitely make it easier.*

[W1-P4]

On the other hand, another participant expressed a preference for completing safety planning all at once:

***Q (Interviewer):** “…if you were interested in creating a safety plan, an additional tool, would you wanna do it sort of in one sitting, or would you wanna kinda space it out over time?”*

***A (Participant):** “I think I’d rather just get it over with.”*

[W2-P7]

W2-P7 viewed the creation of a safety plan as a task that was difficult or aversive in some fashion and preferred to complete the safety plan within a short time frame. Some participants valued the efficiency of safety planning in a single sitting, reinforcing the need for customizable pacing options that accommodate individuals’ preferred working styles and diverse needs.

Finally, participants identified the need to set the timing of messages to align with their own mental state, context, and vulnerability windows. Participants cautioned that poorly timed prompts sent when individuals were unavailable (eg, during class or low-energy periods) could be ineffective and intrusive. Participants stressed the importance of matching system outreach to user readiness and mental state. As W2-P9 pointed out, “I think random prompts might be challenging and less effective…for example, if I’m in class, or if I’m just in a place where I don’t have a lot of mental energy, or I am struggling that day, that’s not the best time to try and write that sort of thing.” Demonstrating the importance of the timing of prompts, 1 participant suggested that users should be able to identify high-risk times, such as evenings or periods of anticipated isolation, so the system can check-in with them during those vulnerable moments. As W1-P4 put it, “If there was some way to have set times when you know you’re likely to be alone like, for example, in the evening…and then maybe it could check up on you in those times.”

#### Customizing Safety Plan Layouts

Participants wanted the ability to actively refine, reorder, and organize the content of their safety plans over time. When thinking about organizing their safety plans, participants shared that changing the order of content was helpful, allowing them to prioritize the most effective coping strategies or key contacts at the top so that essential information remains easily accessible during high-stress moments. As W1-P2 stated, “I think it’s important to think about that when I’m developing the safety plan. And to put that at the beginning so that when I’m really in crisis, I don’t need to read through the whole plan.”

Though less well represented among participants, 1 participant expressed interest in visual customization. This included naming the system, customizing the appearance of intervention messages, and adding meaningful images.

*If maybe there was even a visual personalization, if there was some like you could make the messages look a certain way that you wanted, or if you could maybe even give the automated messaging system a name…so you don’t just have to call it the system or the machine or the name of the app or process, if you could somehow maybe put images in it that you liked or choose a style that it appears in…*

[W1-P4]

W1-P4 highlights that visual customization, while less widely discussed, could further enhance ownership and individualization of the system.

### What Interactions Between Personalization and Customization Do Young Adults Require in a Self-Guided Digital SPI?

Participants indicated that there are some elements of digital safety planning that they would like to have personalized but retain the option of overriding or customizing. We consider these elements to be an interaction between personalized and customized features. Participants detailed that, for these elements, collaboration between the personalized system-level features and the user-level customized features is essential to ensure respect for autonomy while optimizing the usability of a digital safety planning tool.

Participants strongly articulated the importance of a digital safety planning system that suggests certain automated actions while ensuring that the end user can confirm or deny such automated actions. For example, several participants identified that if a digital intervention offered to contact a trusted person or a crisis counselor, the user would need a clear override ability, enabling the system to take action only following active, affirmative consent.

*If the automated system recognizes signs of imminent suicidality in me, if it said something like, “I’m noticing that you’re deep in crisis” or “I’m noticing that you’re having some pretty intense warning signs. Would you like to be connected to a trained crisis counselor? Yes or no?” I text back, like, “Yes, I would.” “Great. I’m connecting you to a therapist.” And then the next text I get is, “Hi. I’m a trained crisis counselor. How can I help you?” Or…sending me a link to a telephone number. Like, “Please call this number when you’re ready.” Or “Would you like one of our counselors to call you?” Or sending me a link to some chat or something. Definitely asking me if I want that, not just forcing it on me.*

[W1-P2]

## Discussion

### Principal Findings

#### Decision Points in Self-Guided Digital Safety Planning

This study aimed to identify key decision points in self-guided digital safety planning and examine how personalization and customization allocate autonomy and decision authority across these points. Participants identified 3 classes of decision points: system-driven personalization, user-driven customization, and interactions between automated guidance and user control. These classes aligned with 3 primary domains of the 5W1H framework. Decisions about what support is delivered were primarily associated with system-driven personalization, including tailored suggestions, prompts, affirmations, and resources. Decisions about when intervention occurs were primarily associated with user-controlled timing, pacing, and responsiveness. Decisions about how users participate and exert control included customization of safety-plan content and layout, as well as the ability to authorize, modify, or decline system-initiated actions. The remaining dimensions, who the system is responding to, where the user is situated, and why the user authorizes or resists an action, functioned as contextual conditions that shaped these decision points across domains rather than as separate categories. This structure highlights how personalization and customization jointly allocate control between users and systems during moments of suicidal distress.

#### Decision Points for What the System Delivers

The *what* domain concerns the content and resources the system should tailor or provide. Participants in the study emphasized the importance of systems automatically providing core safety planning support, particularly crisis resources, coping suggestions, and guidance that can be accessed without requiring complex decision-making during distress. Consistent with prior digital SPIs, participants valued immediate access to crisis resources and individualized coping strategies [36-38]. Our findings extend the existing literature by specifying that the selection and tailoring of crisis resources based on factors such as geography, passively sensed data that might suggest a crisis state, and potential relevance represent a central decision point where systems may be well suited to personalize support and present relevant information to users while remaining nondirective. Critically, decisions about *what* is most relevant are conditioned by the context of *who* the user is at any specific moment, for example, their behavioral state or where they are located, which determines the relevance of crisis resources.

Within this system-level scope, participants highlighted several forms of supportive content that should be personalized. One form of content that participants wanted personalized was reflective prompts. Reflective prompts within a self-guided digital SPI could be designed to help users generate individualized content, increase awareness of symptoms, and identify personalized coping resources. Reflective prompts are commonly supported in DMHIs through free-text engagement [14,39,40]. Prior work has used open-ended reflective prompts that invite and incorporate user-generated input, which supports the personalization of safety plans [39]. Additionally, participants emphasized the value of system-generated example outreach messages. Users of DMHIs benefit from social support features [41,42], and modifiable preprogrammed messages [43] have the potential to reduce cognitive and emotional barriers to social outreach—a protective factor for suicide prevention. These preferences point to specific content decisions that may be well suited for system-level personalization, particularly when the goal is to ease cognitive and emotional demands without directing user behavior during a crisis.

#### Decision Points for When the System Intervenes

The *when* domain concerns message timing, response latency, and whether safety planning occurs in 1 session or across multiple interactions. Participants emphasized the need to consider *when* the system intervenes. Timing decisions shaped whether participants imagined that they could engage with prompts and safety planning tasks given momentary cognitive and emotional capacity. Noting that readiness to engage varies across contexts due to dynamic cognitive and emotional states (the *who* dimension of the 5W1H), participants expressed a preference for flexibility in *when* prompts are delivered and how much time is allowed for response. This preference aligns with prior digital suicide prevention research indicating that effective intervention should identify moments of individual vulnerability and receptivity to optimize timing of intervention components, ensuring that interventions are delivered when users are both in need and willing to engage [11]. Specifically, Bentley et al [38] showed that monitoring real-time suicide risk to dynamically guide the delivery of intervention components increased subsequent use of coping strategies, illustrating that when to intervene is inseparable from *who* is being monitored. Timing decisions, therefore, need to be personalized to an individual’s momentary state.

Beyond moment-to-moment prompting, participants identified the pacing of safety plan completion as a distinct temporal decision point that shaped both effort and engagement. Some preferred completing safety planning in a single session, while others favored distributing tasks across multiple interactions based on attentional and emotional resources. Although prior reviews [41] found that fully unsupervised, self-paced digital interventions may show weaker clinical effects than supervised, clinician-paced models, our findings suggest that pacing itself may be a design decision that can structure and scaffold user effort at key moments, especially in crisis contexts, when cognitive load and capacity may change rapidly.

#### Decision Points for How Users Participate and Control Decisions

The *how* domain concerns how users structure the safety plan and retain authority over system actions. This study identifies user control as a class of decision points in self-guided digital safety planning, specifying where authority should remain with users rather than be assumed by the system. Participants’ preferences for user-controlled decision points reflect young adults’ preferences for self-reliance and autonomy [8,9] (ie, the *why* in the 5W1H) and suggest that customization functions as a structural response to the motivational barriers that can limit engagement with support. Participants described customization operating at multiple levels. At the interface level, users valued the ability to adjust visual presentation and layout, including reordering safety plan components so that the most salient strategies or contacts are immediately accessible during distress. Similar forms of visual and structural customization have been implemented in prior SPI tools and are associated with good usability [18,44].

#### Integration and Dynamic Interaction Between Automated and User-Controlled Decisions

The *why* domain concerns the conditions under which users are willing to authorize or resist system-initiated actions. Participants consistently emphasized that automated personalization in digital SPIs should remain contingent on explicit and revisitable user authorization, reflecting a preference for systems that function as decision aids rather than autonomous actors. This preference aligns with prior work showing that autonomy-related orientations shape help-seeking among young adults, with stronger preferences for self-reliance associated with lower intentions to seek formal mental health support [9]. More broadly, engagement with DMHIs depends on perceived fit as well as autonomy-, privacy-, and confidentiality-related considerations [24].

Building on this foundation, the present findings highlight the importance of integrating system-level and user-level decision points to support both adaptiveness and user control. While prior reviews suggest that personalization and customization can independently promote engagement, usefulness, and perceived relevance [18,24], our results highlight how they function together. Participants described wanting systems that could automatically detect warning signs and generate appropriate suggestions, such as proposing outreach to identified contacts during periods of heightened risk. These system-level decision points were expected to reduce the burden by supplying timely, personalized guidance during distress. At the same time, participants consistently emphasized that such actions should only occur with explicit user permission and the ability to override or revise system behavior.

This interplay underscores that effective digital SPIs must balance automation with user authority, embedding decision points that allow users to retain control over high-stakes actions while still benefiting from adaptive support. This design orientation aligns well with mixed-initiative interaction between users and computer automation [45] and with guidelines for human-AI interactions [46], which conceptualize control as dynamically shared between users and systems depending on contextual uncertainties, tasks, and user capacities. Within this framework, personalization reduces cognitive and emotional load by proactively generating relevant content, while customization preserves agency by enabling users to authorize, modify, or decline system-initiated actions. Importantly, participants viewed this balance as dynamic, requiring systems to support the ongoing renegotiation of control as needs and contexts change. Our findings suggest that at certain key decision points, there must be an integration of automated and user-controlled decisions.

#### Design Implications

These findings suggest 3 design implications for self-guided digital suicide prevention interventions. First, digital suicide prevention interventions should incorporate multistage pacing options, allowing users to complete suicide prevention activities (eg, safety planning tasks) either in a single session or across multiple interactions, based on momentary capacity and context (addressing the *when* domain). Prior research on self-guided DMHIs demonstrates that allowing users to progress at their own speed, rather than following a rigid timeline, supports overcoming barriers to engagement by accommodating fluctuations in emotional and cognitive resources [47,48]. Second, designers should implement structured customization, in which users exercise meaningful control within system-designed, constrained options to preserve agency while preventing excessive complexity or burden (addressing the *how* domain). This can be executed by allowing the users to select their preferences from a list of system-provided options [20]. For instance, users can choose their preferred visual content from a list. Third, systems should adopt adaptive but permissioned automation (addressing the *what*, *who*, and *where* domains). While automated personalization can reduce burden, such automation should be mediated through explicit user consent and ongoing opportunities for adjustment. Decision points related to permissions, such as contacting emergency resources or notifying trusted individuals, should be revisitable and require clear user consent before action.

### Strengths, Limitations, and Future Directions

Importantly, these data reflect the perspectives of young adults actively seeking mental health information and support through MHA. A key strength is recruitment through MHA’s self-screening and suicide-related information pages, enabling direct engagement with end users in real-world help-seeking contexts. Incorporating end users into intervention design and needs assessment enhances ecological validity and supports the development of tools for real-world use.

Several limitations should be considered when interpreting these findings. First, the small sample of 9 participants limits the breadth of perspectives represented and the transferability of the findings. The sample also had limited demographic diversity, including no male participants and limited racial diversity. Second, interview participants were recruited from a parent ARC study [25], and their prior participation in that study may have influenced both their willingness to complete a follow-up interview and the perspectives they shared. Participation in the interviews was voluntary, creating the possibility of volunteer-selection bias. We did not compare interview participants with eligible individuals who did not participate and therefore cannot determine whether the groups differed in their engagement, experiences, or perspectives. Third, 8 of the 9 participants had previously seen a therapist or other mental health professional. Their familiarity with mental health care and safety-related resources may have influenced their preferences and may not reflect the perspectives of young adults without prior service experience. Finally, participants were young adults recruited through MHA who had sought mental health information or support online. The findings may therefore have limited transferability to other age groups, recruitment settings, or individuals who are not actively seeking mental health information.

These limitations highlight the need for further testing and refinement of the decision-point framework across more diverse samples and designs. The framework also provides a foundation for evaluating and optimizing digital SPIs, aligning with just-in-time adaptive intervention principles [11] and the MOST (Multiphase Optimization Strategy) framework [49]. Conceptualizing personalization and customization as decision-based elements enables future microrandomized and optimization studies to examine automation, user override, and consent renewal as manipulable components.

### Conclusions

Self-guided digital suicide prevention interventions should not treat personalization and customization as separate design features but as complementary mechanisms for allocating decision authority between users and systems. The central design challenge is to reduce cognitive and emotional burden through adaptive support while preserving user control over safety-relevant actions. These findings support a model of permissioned automation in which systems can offer tailored guidance and resources while users retain the ability to authorize, modify, or decline actions as their needs and circumstances change.

## Abbreviations

5W1H: Who, What, Where, When, Why, and How
ARC: asynchronous remote community
BDI-II: Beck Depression Inventory–Second Edition
C-SSRS: Columbia Suicide Severity Rating Scale
DMHI: Digital mental health intervention
DSI-SS: Depression Severity Index–Suicidality Subscale
*DSM-5*-CCSI: *Diagnostic and Statistical Manual of Mental Disorders, Fifth Edition*, Cross-Cutting Symptom Inventory
MHA: Mental Health America
MOST: Multiphase Optimization Strategy
SPI: safety planning intervention
STB: suicidal thoughts and behaviors
